# Endothelial to Mesenchymal Transition: An Insight in Atherosclerosis

**DOI:** 10.3389/fcvm.2021.734550

**Published:** 2021-09-17

**Authors:** Qingyan Huang, Yuhong Gan, Zhikang Yu, Heming Wu, Zhixiong Zhong

**Affiliations:** ^1^Center for Precision Medicine, Meizhou People's Hospital, Meizhou Academy of Medical Sciences, Meizhou, China; ^2^Guangdong Provincial Key Laboratory of Precision Medicine and Clinical Translational Research of Hakka Population, Meizhou People's Hospital, Meizhou Academy of Medical Sciences, Meizhou, China; ^3^Guangdong Provincial Engineering and Technology Research Center for Clinical Molecular Diagnostics and Antibody Therapeutics, Meizhou People's Hospital, Meizhou Academy of Medical Sciences, Meizhou, China

**Keywords:** atherosclerosis, endothelial to mesenchymal transition, plasticity of endothelial cells, regulating mechanisms, EndMT-associated marker

## Abstract

Atherosclerosis is a fundamental disease of the cardiovascular system that leads to high morbidity and mortality worldwide. The endothelium is the first protective barrier in atherosclerosis. Endothelial cells have the potential to be transformed into mesenchymal cells, in a process termed endothelial to mesenchymal transition (EndMT). On the one hand, EndMT is known to contribute to atherosclerosis by inducing a number of phenotypes ranging from endothelial cell dysfunction to plaque formation. On the other hand, risk factors for atherosclerosis can lead to EndMT. A substantial body of evidence has suggested that EndMT induces the development of atherosclerosis; therefore, a deeper understanding of the molecular mechanisms underlying EndMT in atherosclerosis might provide insights to reverse this condition.

## Introduction

**Figure 1:**
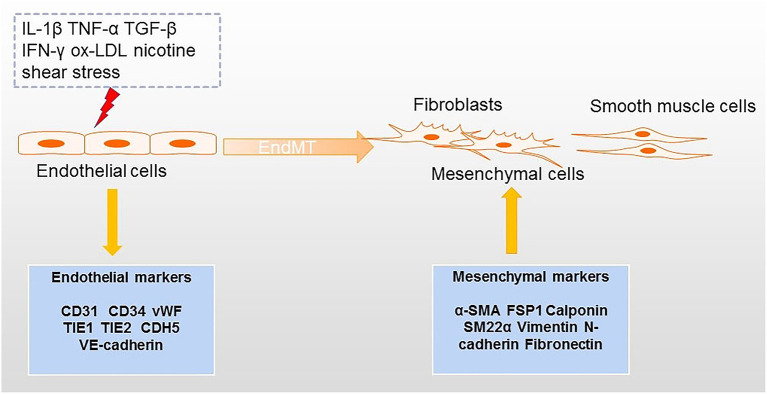
Processes inducing EndMT in atherosclerosis. Factors that can lead to EndMT include several inflammatory cytokines, ox-LDL, nicotine, and shear stress. The transition from endothelial cells to mesenchymal cells (e.g., fibroblasts and smooth muscle cells) is accompanied by downregulation of endothelial markers and upregulation of mesenchymal markers.

Atherosclerosis is a common disease of the cardiovascular system characterized by plaque formation in the artery wall (1). Although some hypothesis based on “inflammation” (2), “lipid” (3) and “immunology” (4) have been proposed to explain the development of atherosclerosis, the pathogenesis of this condition is still not fully understood. Recently, the concept of endothelial to mesenchymal transition (EndMT) has also been put forward to explain the pathophysiological process of atherosclerosis from the perspective of cell trans differentiation (5). EndMT refers to a process in which endothelial cells can be transformed into mesenchymal cells. In this process, endothelial cells acquire the characteristics of mesenchymal cells and feature with loss of cell–cell contact and cell polarity under the condition of biochemical and biomechanical stimulus (6) (Figure 1). The obvious changes involved in EndMT at the molecular level include decreased expression of endothelial cell markers [such as platelet endothelial cell adhesion molecule-1 (CD31), CD34, von Willebrand Factor (vWF), tyrosine kinase with immunoglobulin-like and EGF-like domains 1 (TIE1), TEK receptor tyrosine kinase (TIE2), and vascular endothelial cadherin (CDH5)] and the increased expression of mesenchymal cell markers [such as alpha-smooth muscle actin (α-SMA), ferroptosis suppressor protein 1 (FSP1), Calponin, and smooth muscle 22 alpha (SM22α) (7, 8)]. During EndMT, endothelial cells undergo morphological changes from a cuboidal to a spindle shape. EndMT is a requirement for the formation of endocardial cushions during heart development; cushions resemble the early endothelium (9). EndMT has not only been described in organogenesis, but it has also been implicated in many diseases including pulmonary arterial hypertension (PAH) (10), fibrosis (11), cancer (12), pathological neovascularization (13), and atherosclerosis (14).

In this Review, we focus on the relationship between EndMT and progression of atherosclerosis to help to communicate relevant knowledge for atherosclerosis prevention. We discuss the effects of EndMT and the mechanisms underlying this process in the context of atherosclerosis.

## Triggering EndMT Factors in Atherosclerosis

The endothelium plays an important role in withstanding risk factors against atherosclerosis. Various conditions including sustained inflammation, fluid shear stress, ox-LDL, and smoking can facilitate EndMT (15, 16). Sustained inflammation is a typical pathologic feature of atherosclerosis (17). Inflammatory cytokines including interleukin-1beta (IL-1β), tumor necrosis factor alpha (TNF-α), transforming growth factor-beta (TGF-β), and interferon-gamma (IFN-γ) induce endothelial dysfunction and the acquisition of mesenchymal properties, therefore contributing to atherosclerosis (18–20). Long-term exposure to inflammatory cytokines can induce EndMT by altering the morphology of endothelial cells and EndMT-associated markers (21). In addition to the alteration of expression of endothelial/mesenchymal markers, inflammatory cytokines can also activate the TGF-β pathway and the non-TGF-β pathways (19).

Disturbed blood flow is another pathological characteristic of atherosclerosis. Zhou et al. showed that pulsatile shear stress (PS) exerts an atheroprotective role through the maintenance of endothelial cell homeostasis. In contrast, oscillatory shear stress (OS) causes endothelial cell dysfunction (22). Prior literature has also shown that EndMT can be induced by shear stress (23, 24). In addition, data from next-generation sequencing suggested that PS and OS lead to opposite effects in regulating EndMT gene regulation (25). Notably, expression of the twist family basic helix-loop-helix transcription factor (TWIST) expression was observed under conditions of low shear stress in regions of adult arteries (26). (27) demonstrated that low shear stress can result in the depletion of teneleven-translocation 2 (TET2). Overexpression of TET2 alleviated EndMT in atherosclerosis. Other risk factors associated with atherosclerosis can also lead to EndMT. Oxidized low density lipoprotein (ox-LDL) upregulates EndMT transcriptional factor Snail in human aortic endothelial cells (HAECs), in a on ox-LDLreceptor-dependent manner (LOX-1) (28). Moreover, ox-LDL induces accumulation of reactive oxygen species (ROS) accumulation in cells and synergistically promotes radiation-induced EndMT (29, 30). The effect of ox-LDL on EndMT can be inhibited by vaccarin (31) and naringin (32). Smoking is another known essential risk factor of atherosclerosis. Nicotine treated ApoE^−/−^ mice and human aortic endothelial cells (HAECs) presented with mesenchymal phenotypes, and blocking the nicotine receptor alleviated nicotine-induced EndMT and lesions in mice (33).

## EndMT is Related To Endothelial Cell Plasticity

EndMT is a hallmark of endothelial plasticity. A detailed Review by William C. Aird previously discussed the phenotypic heterogeneity of the endothelium. In the embryonic period, endothelial cell differentiation plays an important role in organ development (34). Endothelial cells differentiate to meet the needs of organ development. For example, multiple lineage hematopoietic progenitors are derived from hemogenic endothelial cells *via* process endothelial-to-hematopoietic transition (EHT) (35). EndMT is essential for heart development. Heart endothelial cells give rise to cardiac fibroblasts and smooth muscle cells, contributing to the formation of the endocardial cushion formation (36). In the adult, disease can occur when homeostasis of endothelial cells is disrupted and these cells remain in an EndMT state.

In atherosclerosis, endothelial cells are exposed to various biochemical and physical stimuli derived from the circulating blood. Low-density lipoprotein (LDL), cholesterol, and wall shear stress may induce EndMT and disrupt endothelial cell homeostasis, leading to endothelial dysfunction and thereby contributing to the development of atherosclerosis. In addition, it has been well documented that inflammatory factors such as IL-1β, TNF-α, TGF-β, and endotoxins can induce endothelial dysfunction *via* EndMT. In inflammation conditions, activation of endothelial cells occurs, leading to phenotypic and molecular changes (16, 37–39). Endothelial cells are heterogeneous and show a high degree of plasticity in both normal and atheromatous conditions. (40) defined eight endothelial cell clusters in the heart and aorta of patients with diabetic atherosclerosis at single cell level, three of which expressed mesenchymal markers, indicating EndMT markers. Further analysis suggested that the proportion of EndMT-derived fibroblast like cells was higher in atherosclerosis group compared to the normal group, with alterations in extracellular-matrix organization, adhesion, and apoptosis.

## EndMT Involves Atherosclerotic Plaque Formation and Instability

The presence of endothelial cell-derived mesenchymal like cells in plaques provides robust evidence of the involvement of EndMT in atherosclerosis. Previous studies demonstrated that atherosclerotic plaques contain mesenchymal cells (including fibroblasts and smooth muscle cells), which regulate inflammation, extra-cellular matrix and collagen production, and plaque structural integrity and play a key functional role in atherosclerosis (41, 42). To investigate the origins of atherosclerosis-associated fibroblasts, (43) examined and confirmed the presence of EndMT-derived fibroblast-like cells present in atherosclerotic lesions through lineage-tracking, suggesting a role of EndMT atherosclerosis development. Matrix metalloproteinases (MMPs) are associated with unstable atherosclerotic lesions (44, 45), and EndMT-derived fibroblast-like cells express higher levels of MMP1, MMP9, and MMP10 compared with normal fibroblasts. In addition, TGF-β signaling, oxidative stress, and hypoxia facilitate endothelial cells conversion to mesenchymal cells and are all hallmarks of atherosclerosis. During EndMT process, active endothelial cells express adhesion molecules, such as intercellular adhesion molecule-1 (ICAM-1) and vascular cell adhesion molecule-1 (VCAM-1), which enhance monocyte, leukocyte, and macrophage recruitment and infiltration (46). Interestingly, (47) found evidence for a crosstalk between macrophages and EndMT: macrophages in atherosclerotic lesions *in vivo* upregulate the expression of mesothelial markers, promoting EndMT, and, conversely, EndMT cells impact the function, such as the capacity of lipid uptake, and phenotypes of macrophages. In addition to *in vivo* and *in vitro* studies, single cell sequencing technology has been helpful to further our understanding of the landscape and pathophysiology of human atherosclerotic plaques. Recently, a study identified 14 cell populations including endothelial cells, smooth muscle cells, mast cells, B cells, myeloid cells, and T cells and identified multiple cellular activation states in plaques (48). One of the identified subclasses of endothelial cells expressed the smooth muscle cell markers such as alpha-actin 2 (ACTA2), notch receptor 3 (NOTCH3), and myosin heavy chain 11 (MYH11), suggesting that this subtype was undergoing EndMT and providing additional evidence on plasticity of endothelial cell plasticity. Altogether, these findings confirm that EndMT is closely associated with plaque initiation and development.

## Mechanisms Regulating EndMT in Atherosclerosis

**Figure 2:**
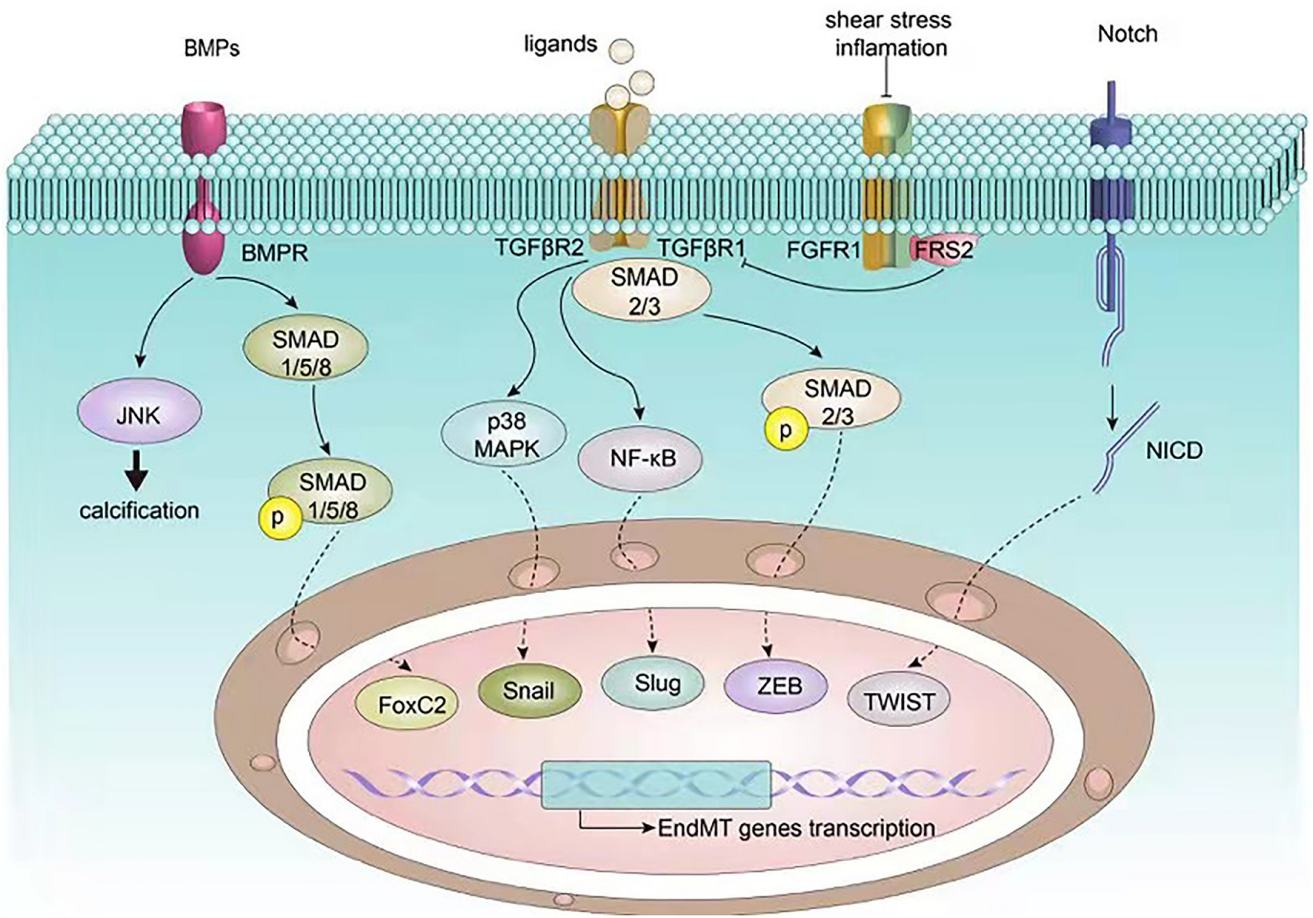
Signaling pathways involved in EndMT in atherosclerosis. EndMT related transcription factors such as Snail, Slug, ZEB, TWIST, and FoxC2 are governed by upstream signalings including BMP, TGF-β, FGF, and Notch, which in turn regulate endothelial and mesenchymal gene expression, ultimately inducing EndMT.

A substantial body of research pinpoints to several features of atherosclerosis that can lead to EndMT *via* several signaling pathways (49, 50). The TGF-β signaling pathway is a canonical pathway modulating EndMT, which has been shown to crosstalk with other pathways including the fibroblast growth factor (FGF), Notch, and bone morphogenetic protein (BMP) pathways (Figure 2). In addition, non-coding RNAs also paly an essential role in EndMT. We next discuss the mechanisms underlying EndMT in the context of atherosclerosis.

## TGF-β Signaling Pathway

The role of TGF-β signaling pathway on EndMT has been extensively studied. In brief, the TGF-β family ligands bind to type I receptors and type II receptors, phosphorylating and thereby activating the transducer small mother against decapentaplegic (SMAD). Nuclear import of active SMAD transmits regulates gene transcription (51). In addition, TGF-β receptors can also activate other signaling pathways. Specifically, TGF-β signaling can be divided into SMAD-dependent and non-SMAD-dependent pathway (52). Growing evidence demonstrated the role of TGF-β signaling pathway in regulating cell proliferation, differentiation, adhesion, migration, and apoptosis not only in both embryonic development and the pathology of human disease (53). (14) showed that risk factors of atherosclerosis such as oscillatory shear stress and inflammation-induced loss of fibroblast growth factor receptor 1 (FGFR1) expression can activate TGF-β signaling and contribute to EndMT. FGFR1 depletion induces EndMT by upregulation of smooth muscle markers and mesenchymal markers which are also targets of TGF-β (54). Although the crucial role of TGF-β in EndMT has been well established, the different roles of the various isoforms of TGF-β are less known. To this aim, Sabbineni et al. (55) compared the effect of three isoforms of TGF-β (TGF-β1–3) on EndMT and found that TGFβ2 was the one most associated with EndMT. In addition, TGFβ2 is required for epithelial-to-mesenchymal cell transformation during endocardial cushion (56). Experiments treating human microvascular endothelial cells (HMECs) with different TGFβ isoforms indicated that only TGF-β2 substantially increased Smad2/3, p38 mitogen-activated protein kinase (MAPK), and mesenchymal transcription factors Snail and forkhead box protein C2 (Foxc2). Furthermore, TGF-β2 and IL-1β co-stimulate EndMT to activate nuclear factor-kappaB (NF-κB) (19).

Given the critical role of TGF-β in regulating EndMT, TGF-β inhibition may be an approach to reverse EndMT. Knockout of endothelium-specific TGF-β receptor relieved EndMT and kidney fibrosis (57). However, TGF-β exhibits potent regulatory functions, and inhibition of this pathway might also result in unwanted side effects.

Altogether, this evidence suggests that TGF-β may also be a promising therapeutic target for atherosclerosis, and further studies to confirm this hypothesis and assess safety are needed.

### BMP Signaling Pathway

Bone morphogenetic proteins (BMPs) belong to TGF-β signaling pathway superfamily. The crosstalk of TGF-β/BMP signaling is well understood (58). More than 20 BMPs with different functions have been identified so far (59). The BMP ligand-receptor interaction induces SMAD1/5/8 phosphorylation to mediate downstream signaling (60). BMPs bind to two different receptors to mediate signal transduction through SMAD-dependent and SMAD-independent pathways (61). (62) showed down regulation of BMP type II receptor BMPR2 in pro-inflammatory-induced EndMT, with enhanced BMP-9-induced osteogenic differentiation, which leads to a decrease in c-Jun N-terminal kinase (JNK) signaling, thus contributing to calcification. Similarly, another study suggested a protective role for BMPR2 in endothelial cell homeostasis, in particular by balancing BMP/TGF-β signaling to protect cells from increased responses toward TGF-β (63). In addition to BMPR2, BMP6 also has the ability to induce osteogenic differentiation and mineralization consistent with EndMT. Activation of reactive oxygen species (ROS) is required for BMP6 to regulate osteogenic genes, osteogenic differentiation, and calcification (64). Furthermore, (29) found that brain and muscle ARNT-like protein-1 (BMAL1) suppressed ROS-induced EndMT through BMP signaling, therefore inhibiting atherosclerosis plaque progression.

### Notch Signaling Pathway

Notch signaling pathway includes 4 receptors (Notch1–4) and 5 ligands (Delta-like-1, 3, 4, and Jagged1–2) (65). Notch receptor-ligand binding generates Notch intracellular domain (NICD) which is translocated to the nucleus where NICD binds to the transcription factor RBPJK/CSL to regulate target gene expression and thus cell fate specification (66). Within the cardiovascular system, Notch signaling is implicated in both development, such as cardiac valve formation, and pathological process, such as response to vascular injury (67, 68). It was observed that Notch receptors and ligands are expressed in the vasculature (69). Notch activated endothelial cells show the characteristics of EndMT including downregulation of endothelial markers and upregulation of mesenchymal markers (70). In addition, Notch activation induces Slug overexpression in endothelial cells which is associated with a loss of the endothelial phenotype (71). Notch signaling contributes to EndMT independently of or synergistically with TGF-β. TGF-β1 induces upregulation of several Notch components including Jagged-1, the receptor Notch-1, N1ICD, recombination signal binding protein J kappa (RBPJK), as well as target genes hairy enhancer of split-1 (Hes-1) and Hes-5(72). Activation of Notch signaling pathway *in vitro* induced EndMT by increasing the expression of vascular endothelial (VE)-cadherin and overexpression of α-SMA, whereas inhibition of Notch signaling pathway with gamma-secretase inhibitors (GSI) attenuated the development of atherosclerotic lesions (73).

### Non-coding RNAs

Non-coding RNAs including microRNAs (miRNAs), long non-coding RNAs (lncRNAs), and circular RNAs (circRNAs) are involved in the regulation the process of EndMT. (74) have provided a comprehensive description of the non-coding RNAs known to be involved in EndMT regulation. In this Review, we focus on non-coding RNAs influencing EndMT and atherosclerosis progression (Table 1). MiRNAs are a class of non-coding RNAs with a length of 20–40 nucleotides, which suppress target mRNAs by binding to their 3'-UTR (75). miR-449a was highly expressed in ApoE^−/−^ diabetic mice and modulated EndMT by increasing the expression of mesenchymal cell markers and reducing E-cadherin which interacts with adiponectin receptor 2 (AdipoR2) in lipid rafts. ApoE-/- diabetic mice treated with an antagonist of miR-449a showed reduction of atherosclerotic lesions (76). miR-374b induces EndMT by targeting mitogen-activated protein kinase 7 (MAPK7), which is decreased in the atheroprone hyperplastic regions and is known to be inhibit EndMT. In the TGF-β treated endothelial cells, the increased level of miR-374b was counteracted by an inhibitor of ALK5 (SB431542). In addition, silencing miR-374b targets by means of short hairpin RNAs (shRNAs) specifically decreased MAPK7 signaling members, and increased expression of endothelial markers VE-cadherin and endothelial nitric oxide synthase (eNOS), and of the mesenchymal markers SM22α and Calponin (77). In a recent study, high expression of miR-122 was observed in both ApoE^−/−^ mice and *in vitro* EndMT models, and miR-122 inhibition in ApoE-/- mice reduced the progression of plaque formation. Similarly, inhibiting miR-122 could reverse the EndMT phenotype induced by H_2_O_2_, whereas, silencing of the miR-122 target neuronal PAS domain protein 3 (NPAS3) gene abolished EndMT reversal. Therefore, this study suggests that miR-122 promotes plaque formation *via* NPAS3 mediated EndMT and could be a new therapeutic target in atherosclerosis (78).

**Table 1:** Non-coding RNAs regulating EndMT in atherosclerosis.

**Non-coding RNAs**	**Targets**	**Regulation**	**Models**	**Year**	**Reference**
miR-449a	AdipoR2	upregulation	ApoE^−/−^ diabetic mice Human carotid atherosclerotic plaque HUVECs	2019	(76)
miR-374b	MAPK7	upregulation	Human coronary arteries Pigs HUVECs	2019	(77)
miR-122	NPAS3	upregulation	ApoE^−/−^ mice HAECs HUVECs	2021	(78)
H19	TET1/TGFβR2/TSP1	upregulation	H19 KO mice Primary mouse pulmonary endothelial cells HAECs HUVECs	2010	(83)
MALAT1	Wnt/β-catein	upregulation	ApoE^−/−^ mice HUVECs	2019	(85)
LIC00657	miR-30c-5p/Wnt7b/β-catenin	upregulation	Atherosclerosis patients serum HUVECs	2020	(86)
LncRNA ZFAS1	miR-150-5P/Notch3	upregulation	ApoE^−/−^ mice HUVECs	2021	(89)

*List of non-coding RNAs regulating EndMT associated with atherosclerosis including miRNA and lncRNAs. AdipoR2, Adiponectin receptor 2; MAPK7, Mitogen-activated protein kinase 7; NPAS3, Neuronal PAS Domain Protein 3; TET1, Tet Methylcytosine Dioxygenase 1; TGFβR2, TGF-βreceptor 2; TSP1, Thrombospondin 1; MALAT1, Metastasis-associated lung adenocarcinoma transcript 1; HUVECs, Human umbilical vein endothelial cells; HAECs, Human Aortic Endothelial Cells*.

LncRNAs are non-coding RNAs with a length of more than 200 nucleotides that have recently emerged as important regulators in development and disease (79). So far, although more than 5000 lncRNAs have been identified (80), few of them have been implicated in EndMT. LncRNA H19 is increased in aortic tissues and is associated to the extent of cardiovascular disease in a model of atherosclerosis (81, 82). H19 upregulates TGF-β receptor 2 (TGFβR2) and thrombospondin 1 (TSP1) *via* let-7/TET1, and therefore has the potential to model EndMT markers including Slug, SM22-α, Vimentin, and fibronectin1 (FN1) (83). LncRNA metastasis-associated lung adenocarcinoma transcript 1 (MALAT1) has been shown to play a critical role in cardiovascular disease (84). Li et al. (85) observed an increasing expression of MALAT1 in atherosclerotic mice and human umbilical vein endothelial cells (HUVECs) treated with ox-LDL which also showed CD31 and vWF downregulation and α-SMA and vimentin overexpression. MALAT1 promotes β-catenin translocation to nuclear translocation and enhances EndMT induced by ox-LDL in a MALAT1/Wnt/β-catenin dependent manner. Interestingly, LINC00657 was found to be overexpressed in the serum of atherosclerosis patients and in HUVECs treated with ox-LDL. LINC00657 has a similar effect on inducing EndMT. LINC00657 promotes EndMT by acting as a sponge for miRNA-30c-5p and by positively regulating Wnt7b/β-catenin activation (86). LncRNA ZFAS1 is another contributor of atherosclerosis (87, 88). Results from *in vivo* and *in vitro* atherosclerosis models suggested that ZFAS1 triggered EndMT *via* inhibition of miR-150-5p, hence increasing the expression of Notch3, an active regulator of EndMT (89). Currently, the function of lncRNAs and circRNAs in regulating EndMT in atherosclerosis is largely unknown. CircRNAs associated with EndMT have been reported in many diseases including neuro inflammatory disorders (90), bladder carcinoma (91), pulmonary disease (92), and ischaemic stroke (93). Additional research to clarify the role of lncRNAs and circRNAs in disease is needed.

### Preventing EndMT as a Potential Approach to Treat Atherosclerosis

Given the role of EndMT in modulating atherosclerosis, disruption of the EndMT might be a therapeutic option for treating atherosclerosis. Indeed, some compounds and clinical drugs may have protective effect on atherosclerosis by inhibiting EndMT. RGFP966 is an inhibitor of histone deacetylase 3 (HDAC3), an important regulator of cardiovascular diseases which was found to be upregulated in atherosclerotic lesions (94), and can reduce atherosclerotic lesions by inhibiting EndMT in the aortic root (95). Icariin, a compound derived from *Epimedium*, inhibited ox-LDL-induced EndMT *via* H19/miR-148b-3p/ELF5 (E74-like factor 5). Icariin induced H19 overexpression and led to an attenuation of the EndMT process, exerting a protective effect in atherosclerosis (96). Recently, simvastatin, a clinical lipid-lowering drug, was shown to inhibit EndMT (25). A study by (25) has shown that simvastatin can inhibit EndMT *via* upregulation of KLF4/miR-483 axis in HUVECs. Additionally, simvastatin attenuated 1-Palmitoyl-2-(5-oxovaleroyl)-sn-glycero-3-phosphocholine (POVC) inducing EndMT, by suppressing oxidative stress and TGF-β/SMAD signaling, suggesting that simvastatin could potentially be used in treating atherosclerosis (97). Altogether, these findings suggest the therapeutic potential of EndMT disruption in atherosclerosis. EndMT is a complex process resulting from the action of many factors, ranging from signaling pathways to non-coding RNAs. However, effective drugs to reverse EndMT are still lacking. Moving forward, single-cell/high-throughput sequencing technology might provide helpful insights to uncover EndMT associated targets for the treatment of atherosclerosis.

## Highlighting the Progression of EndMT in Atherosclerosis

The studies by Chen et al. (14) and Evrard et al. (43) provide strong evidence for the involvement of EndMT in atherosclerosis. EndMT drives the initiation of atherosclerosis by accelerating plaque growth and instability of plaque. Previous research has shown that EndMT is regulated in a number of signaling pathways, in particular the canonical TGF-β signaling, and several atherosclerosis risk factors, e.g., inflammation, shear stress, ox-LDL, and smoking. Conversely, FGF signaling plays a protective role on EndMT. Remarkably, several non-coding RNAs were reported to modulating EndMT, offering therapeutic application to treat atherosclerosis (74). Epigenetic mechanisms involved in EndMT regulation are poorly understood. Histone deacetylases (HDACs) play a role in EndMT, in particular HDAC3 and HDAC9, and promoted atherosclerosis progression (98). Overall, the molecular mechanisms underlying EndMT remain largely unknown, and additional research is needed to discover new targets that can be explored in reverse atherosclerosis.

## Conclusions

EndMT is associated with the formation of atherosclerotic lesions. Therefore, reversing or inhibiting EndMT might help to prevent the development or progression of atherosclerosis. *In vivo*, suppression of EndMT shows promising effects in alleviating atherosclerosis. However, animal and cell models of atherosclerosis present many limitations, and the study and detection of EndMT in humans offer great challenges. More research is needed to understand the role of EndMT in atherosclerosis to ultimately offer new insights for the treatment of atherosclerosis.

## Author Contributions

QH, HW, and ZZ contributed to the conception of the study, performed the literature search, and wrote the manuscript. QH, YG, and ZZ edited the manuscript. ZY and YG assisted in the literature search and critically revised the article for important intellectual content. All authors have read and approved the final manuscript.

## Funding

This study was supported by the Guangdong Provincial Key Laboratory of Precision Medicine and Clinical Translation Research of Hakka Population under Grant 2018B030322003; the Science and Technology Program of Meizhou under Grant 2019B0202001.

## Conflict of Interest

The authors declare that the research was conducted in the absence of any commercial or financial relationships that could be construed as a potential conflict of interest.

## Publisher's Note

All claims expressed in this article are solely those of the authors and do not necessarily represent those of their affiliated organizations, or those of the publisher, the editors and the reviewers. Any product that may be evaluated in this article, or claim that may be made by its manufacturer, is not guaranteed or endorsed by the publisher.
